# Effect of the COVID-19 pandemic on life expectancy in Australia, 2020-22

**DOI:** 10.1093/ije/dyad121

**Published:** 2023-09-25

**Authors:** Tim Adair, Brian Houle, Vladimir Canudas-Romo

**Affiliations:** Nossal Institute for Global Health, Melbourne School of Population and Global Health, University of Melbourne, Melbourne, VIC, Australia; School of Demography, Research School of Social Sciences, Australian National University, Canberra, ACT, Australia; MRC/Wits Rural Public Health and Health Transitions Research Unit (Agincourt), School of Public Health, Faculty of Health Sciences, University of the Witwatersrand, Johannesburg, Gauteng, South Africa; School of Demography, Research School of Social Sciences, Australian National University, Canberra, ACT, Australia

**Keywords:** COVID-19, mortality, life expectancy, Australia, causes of death

## Abstract

**Background:**

Australia provides a valuable international case study of life expectancy during the pandemic. In contrast to many other countries, it experienced relatively stringent restrictions and low COVID-19 mortality during 2020–21, followed by relaxation of these restrictions when high vaccination rates were achieved. This study measures Australia’s life expectancy trends and the contributions of age group and causes of death, during the pandemic.

**Methods:**

Trends in life expectancy at birth in Australia and its states and territories were measured from 2020 to 2022. The contributions of age group and cause of death to these trends were measured using decomposition methods. Life expectancy was compared with other high-income countries.

**Results:**

Australia’s life expectancy fell by more than half a year in 2022, following a sharp increase in 2020 and moderate decline in 2021. For the 3 years 2020 to 2022, life expectancy was 0.13 years (95% confidence interval 0.07-0.19) higher for males and 0.09 years (0.03-0.14) higher for females versus 2017–19. Australia’s life expectancy increase in 2020 was larger than that in the vast majority of other high-income countries, but its decline in 2022 was greater than in other countries whose life expectancy rose in the first year of the pandemic. The small negative contribution of COVID-19 deaths to life expectancy in Australia was more than offset by lower non-communicable disease mortality. There were only small differences in life expectancy change between the states with the most stringent restrictions (Victoria and New South Wales) and the rest of Australia.

**Conclusions:**

Australia’s life expectancy trends during 2020–22 were relatively favourable compared with other high-income countries, with the exception of its sharp decline in 2022 once restrictions were loosened.

Key Messages

- This study shows that life expectancy in Australia during the COVID-19 pandemic from 2020 to 2022 was higher (0.13 years male, 0.09 years females) than before the pandemic in 2017–19, despite a sharp decline in 2022 following relaxation of pandemic restrictions.
- Australia’s life expectancy during the pandemic was relatively favourable compared with other high-income countries, except for its decline in 2022.
- COVID-19 mortality only made a small negative contribution to life expectancy of 0.24 years for males and 0.19 years for females, which was more than offset by lower non-communicable disease mortality.
- Life expectancy trends in the states of Victoria and New South Wales, which had the most stringent restrictions and where outbreaks of the virus were worse, were similar to the rest of Australia.
- Australia’s favourable life expectancy during the pandemic can be attributed to its relatively stringent lockdowns and opening up of most restrictions only once vaccination rates had reached a high level.

## Background

The COVID-19 pandemic has had a profound impact on life expectancy globally. In 2020, reductions in life expectancy occurred in the vast majority of high- and upper-middle income countries, exceeding 2 years in Russia and the USA and 1 year in several other countries.^1–3^ In 2021, however, there was greater heterogeneity in life expectancy trends with respect to 2020: some Eastern European countries experienced larger declines, the USA declined by a further 0.2 years and many Western European countries recovered, in some cases all of their 2020 declines.^3^ These declines in life expectancy occurred following several decades of increases in most countries after World War II, representing the largest global mortality shock experienced since that period.^3^

Australia, by contrast, was one of few high-income countries whose life expectancy increased in 2020, rising by 0.7 years for both females and males—the largest increase since the early 1990s.^1^^,^^2^^,^^4^ Australia’s Federal, State and Territory Governments introduced a wide range of restrictions to control the spread of the virus, which have been rated as the seventh most stringent restrictions among 34 high-income countries.^5–7^ International borders were closed from 19 March 2020 to all people aside from Australian citizens, permanent residents and their families, who had to serve a period in quarantine upon re-entering the country.^8^ All of the country’s eight states and territories implemented lockdowns from this time, including closure of schools and non-essential businesses, with restrictions on gatherings and visits to hospitals and aged-care facilities. These restrictions were most stringent in the second most populous state of Victoria, where outbreaks of the virus led to almost 90% of Australia’s 906 COVID-19 deaths during 2020 occurring there.^9^ In 2021, Australia again avoided significant mortality due to COVID-19, experiencing a total of 1349 deaths which occurred almost entirely in the most populous states of New South Wales (NSW) and Victoria, mostly in the second half of the year during the Delta Wave.^10^^,^^11^ The spread of the Delta strain saw these two states and the Australian Capital Territory (ACT) adopt the most stringent restrictions in 2021.^8^ That year also saw the roll-out of the COVID-19 vaccination in Australia, mostly in the second half of the year, with 79% of the population aged 70 years and above fully vaccinated by the beginning of October and 100% by the end of the year.^12^ The continued low COVID-19 mortality meant that age-standardized death rates in Australia during 2020–21 were 5.9% lower compared with 2015–19.^5^

In early 2022, restrictions gradually lifted after vaccination targets were reached, with schools returning to normal operations, international borders re-opening to fully vaccinated international visitors in February and Western Australia (WA) the last state to re-open its state border in March. Mortality increased in 2022, and it has been estimated that excess deaths from all causes in Australia in 2022 were 12% higher (or 20 200) compared with what was expected in the absence of the pandemic, in contrast to just 2% in 2021, with just over half of excess deaths reported as due to COVID-19.^13^

The trend in life expectancy in Australia after the loosening of restrictions is a particularly interesting international case study, given how stringent these restrictions were and the low mortality in 2020–21. This study measures trends in Australia’s life expectancy during the pandemic, including comparison with other high-income countries and the contributions of age group and causes of death to these changes. These analyses include state and territory comparisons, given the different stringency of restrictions implemented across each jurisdiction. We also assess actual versus forecast life expectancy based on pre-pandemic mortality trends.

## Methods

In this study we analysed all-cause and cause-specific mortality data in Australia from 2017 until 2022. All-cause mortality and cause of death data were available from the Australian Bureau of Statistics (ABS) from existing publications (2017–20) and customized data extractions (2021–22) by sex and 5-year age group.^9^^,^^14–16^ Cause of death data were purchased from the ABS for 11 causes of death that were either leading causes or those causes expected to be most affected by the pandemic. Data from the World Health Organization (WHO) Mortality Database were used to provide additional data by cause and age in 2017–20 not available in ABS publications.^17^ All-cause mortality data in 2022 were adjusted for late registration using monthly reports of deaths by the ABS; this increased the number of deaths by 0.6%.^18–29^

Cause of death data for 2022 were available for the 87% of deaths that were doctor-certified but not for the 13% of deaths that were coroner-certified. We estimated cause-specific coroner-certified deaths in 2022 by applying the cause distribution of age-sex-specific coroner-certified deaths in 2021 to the age-sex-specific coroner-certified deaths in 2022.^16^ The reported doctor-certified and estimated coroner-certified cause-specific deaths for 2022 were then summed to estimate the total cause-specific deaths for that year. We separately adjusted the estimated COVID-19 deaths upwards using a sex-specific adjustment factor to ensure they equalled the total sex-specific COVID-19 deaths (doctor- plus coroner-certified) reported by the ABS for 2022.^30^ Next, estimated sex-specific deaths from each other cause were reduced proportionally to offset the increase in COVID-19 deaths. These final cause estimates were used to calculate cause-specific mortality fractions by sex and age for 2022.

State and territory mortality data were obtained from the same sources as for Australia.^9^^,^^14–16^ National and state/territory population data were also obtained from the ABS.^31^ State/territory deaths were also adjusted for late registration. For states/territories, we analysed two causes: COVID-19 deaths and all other causes combined. The doctor-certified COVID-19 deaths by age and sex were adjusted so that they equalled the ABS-published total COVID deaths by state/territory and sex.^30^^,^^32^ Further information about data sources and adjustments are shown in the Supplementary Material (available as Supplementary data at *IJE* online).

Using these data, we calculated age-specific death rates using standard procedures of smoothing the oldest ages 85 and above with a logistic model and derived life tables.^33^ From those life tables, life expectancy at birth by sex, nationally and by state/territory for 2017–19, 2020, 2021 and 2022, as well as 2020–2022 to assess the first 3 years of the pandemic, were obtained. The years 2017–19 were used to measure pre-pandemic mortality to reduce any annual variation in life expectancy. Trends in life expectancy at age 60 years were also assessed as an alternative measure of the impact of the pandemic on mortality; 95% confidence intervals were calculated using Poisson bootstrap sampling of age-specific death counts. We measured the contribution of age and cause to changes in life expectancy at birth using standard life expectancy decomposition techniques.^34^ In our main analysis, we examined the following underlying causes of death (with International Classification of Diseases, 10th Revision codes): COVID-19 (U07.1, U07.2), ischaemic heart disease (IHD, I20-I25) and cerebrovascular diseases (I60-I69) combined, respiratory diseases (combining influenza (J09-J11), pneumonia (J12-J18), other respiratory diseases (J00-J06, J20-J98)), cancers (C00-D48) and all other causes combined (more detailed cause-specific analyses are included in the Supplementary Material, available as Supplementary data at *IJE* online). For states and territories, we only analysed COVID-19 and all other causes combined.

Results for Australia were contrasted with those for selected other high-income countries to compare their mortality experience during the pandemic.^3^^,^^35^ We also calculated how many additional deaths Australia would have experienced in 2020 if it had experienced the same age-specific death rates as each of 27 countries in the Human Mortality Database.^35^ For an alternative assessment of trends in Australia’s life expectancy during the pandemic, we also compared with life expectancy forecast using the Lee–Carter based on 2010–19, again using Human Mortality Database data.^35^^,^^36^ All analyses were conducted using R Statistical Software v4.2.2.^37^

## Results

**Figure 1.**
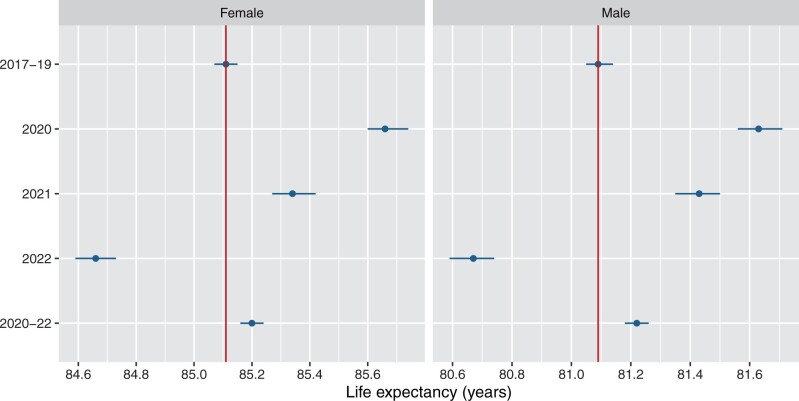
Life expectancy at birth by sex and year(s), Australia. Bars show 95% confidence intervals and the vertical line (red) corresponds to life expectancy in 2017–19. Source: author calculations based on Australian Bureau of Statistics data^14–16^^,31^

Life expectancy increased by more than half a year (males: 0.54 years, 95% confidence interval 0.45 to 0.63; females: 0.55, 0.47 to 0.64) in Australia in 2020 versus 2017–19 (Figure 1 and Supplementary Figure S1, available as Supplementary data at *IJE* online). In 2021, it then declined 0.21 years (−0.32 to −0.10) for males and 0.32 years for females (−0.42 to −0.23) to a level still above 2017–19 for each sex. In 2022, life expectancy declined 0.76 years for males (−0.87 to −0.65) and 0.68 years for females (−0.77 to −0.59). However, for 2020 to 2022, life expectancy was 0.13 years higher for males (0.07 to 0.19) and 0.09 years higher for females (0.03 to 0.14) than in 2017–19. The trend in life expectancy at age 60 years was very similar as for life expectancy at birth, with the change being within 0.10 years of life expectancy at birth for each comparison (Supplementary Table S1, available as Supplementary data at *IJE* online).

**Figure 2.**
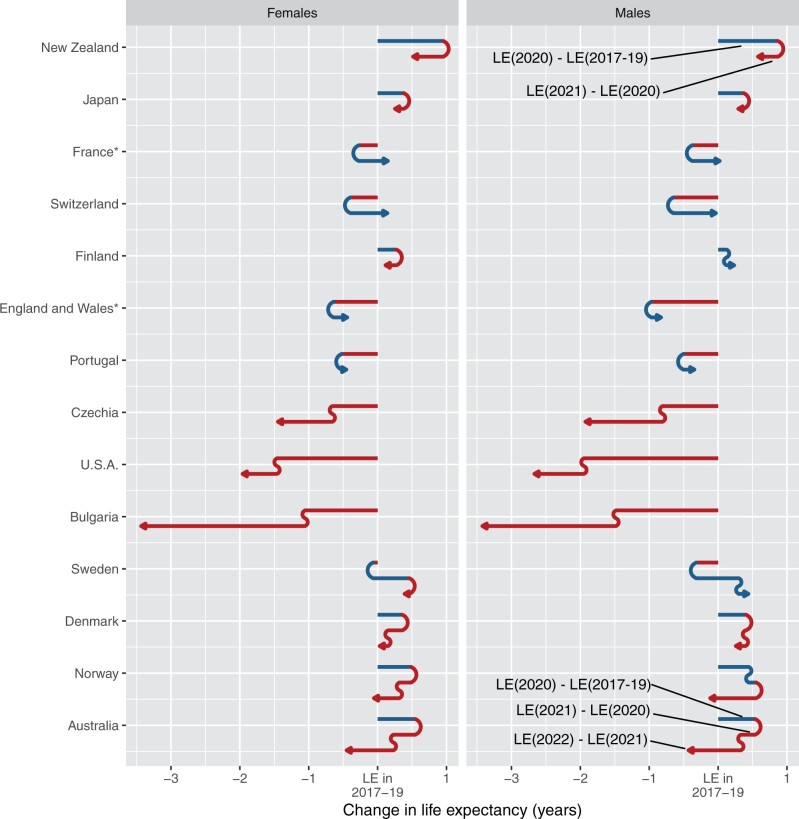
Change in life expectancy at birth by sex compared with 2017–19, Australia 2020–2022 and selected countries 2020–2021 or 2020–22. Bars in the direction of left (red) correspond to declines in life expectancy, and bars in the direction of right (blue) to increase. When similar trends follow over years, then the colour remains but a curve is included. *2017–20 data from Human Mortality Database, 2021 data from Scholey *et al.* (2022). For other countries, 2017–22 data from Human Mortality Database or Australian Bureau of Statistics (Australia). Source: author calculations based on data from Human Mortality Database, Australian Bureau of Statistics and Scholey *et al*. (2022)^3^^,^^14–16^^,^^31^^,^^35^

The increase in life expectancy in Australia in 2020 compared with 2017–19 was markedly different from declines in the USA (−2.0 years males, −1.5 years females), Bulgaria (−1.5 years males, −1.1 years females), England and Wales (−0.6 years females, −1.0 years males) and France (−0.3 years females, −0.4 years males) (Figure 2). The rise in life expectancy in Australia was higher than the increase in Japan, Norway, Denmark and Finland (+0.1 to +0.5 years males and females) but smaller than in New Zealand (+0.9 years males, +1.0 years females). For all but two (Hong Kong and Japan) of 27 other high-income countries, Australia would have had more deaths in 2020 if it had experienced the other country’s death rates (Supplementary Table S2, available as Supplementary data at *IJE* online). Australia would have experienced only slightly more deaths in 2020 if it experienced the same death rates as the Republic of Korea (2% more), and moderately more for New Zealand and Norway (each 10% more). In contrast, if Australia had the same death rates as Bulgaria or Hungary it would have experienced more than double the deaths in 2020, whereas if it had the same death rates as the USA it would have experienced 65% more deaths.

In 2021, life expectancy worsened further in Bulgaria (−1.9 years males, −2.4 years females) and the USA (−0.7 years males, −0.5 years females), there was a slight improvement for England and Wales, and France and Switzerland returned to approximately 2017–19 levels (Figure 2). In Sweden which, unlike other Nordic countries, experienced a decline in life expectancy in 2020, there was an increase in life expectancy in 2021 to a level above the pre-pandemic figure. In countries other than Australia which experienced a rise in life expectancy in 2020, there was a subsequent decline by the latest year of data (2021 or 2022), except for Finland males, which resulted in life expectancy either remaining close to (Norway) or above the 2017–19 level (e.g. New Zealand, Japan, Denmark males). For countries with data to 2022, the post-2020 declines in life expectancy were smaller than those for Australia.

**Figure 3.**
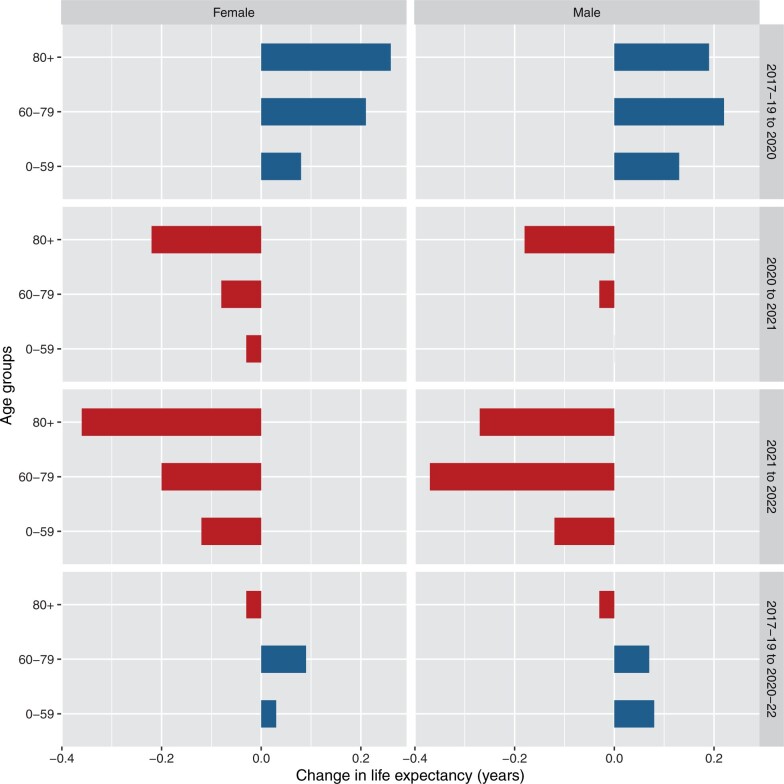
Age decomposition of the change in life expectancy at birth by sex and year(s), Australia. Bars to the left (red) correspond to contributions to the decline in life expectancy, and bars to the right (blue) to increase. Source: author calculations based on Australian Bureau of Statistics data^14–16^^,31^

The increases in life expectancy in Australia in 2020 were highest in the 80 years and above age group for females and the 60–79 years age group for males (Figure 3). For each sex they were lowest in the 0–59 years age group, especially for females. In 2021, most of the declines in life expectancy were in the 80 years and above age group (69% of declines for females, 86% for males). In 2022 just over half of the decline in life expectancy for females was in the oldest age group, but there was a slightly larger contribution from the 60–79 years age group for males. Over 2020 to 2022, the 80 years and above age group contributed a negligible decrease for females and males, whereas for females most of the increase came from the 60–79 years group and for males about an equal amount from the 60–79 and 0–59 years groups.

**Figure 4.**
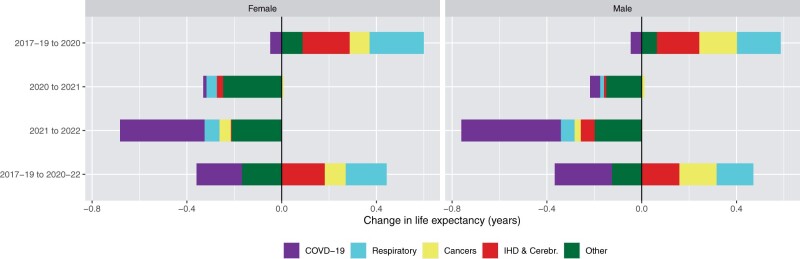
Cause decomposition of the change in life expectancy at birth by sex and year(s), Australia. Bars to the left (negative) correspond to contributions to decline in life expectancy, and to the right (positive) to increase. Source: author calculations based on Australian Bureau of Statistics and World Health Organization data^9^^,^^14–17^^,31^

The rise in life expectancy in Australia in 2020 was caused by declines in mortality from respiratory conditions, IHD and cerebrovascular diseases, cancers and other causes (Figure 4, Supplementary Figure S2, available as Supplementary data at *IJE* online). There was a slight negative contribution to life expectancy by COVID of less than 0.05 years. Most of the decline in 2021 was from other causes (especially in the 80 years and above group—see Supplementary Figure S3, available as Supplementary data at *IJE* online) and less so from respiratory diseases and COVID-19 (again less than 0.05 years). In 2022, COVID-19 contributed to 52% of the decline in life expectancy for females and 55% for males, with most of the remaining decline due to external and other causes and a small negative contribution from IHD and cerebrovascular diseases, cancers and respiratory diseases. This negative contribution of COVID-19 was greatest in the oldest age group (Supplementary Figure S3, available as Supplementary data at *IJE* online). Over the period 2020 to 2022, COVID-19 contributed to 0.24 years of decline in life expectancy for males and 0.19 years for females—the other contributors to declines were dementia, diabetes and external and other causes (combined), although to a lesser extent than COVID-19. Notably, respiratory diseases had a combined positive contribution of 0.16 years for males and 0.17 years for females, only slightly less than the negative contribution of COVID-19. Cancers, IHD and cerebrovascular diseases contributed to a combined increase in life expectancy of 0.32 years for males and 0.27 years for females.

**Figure 5.**
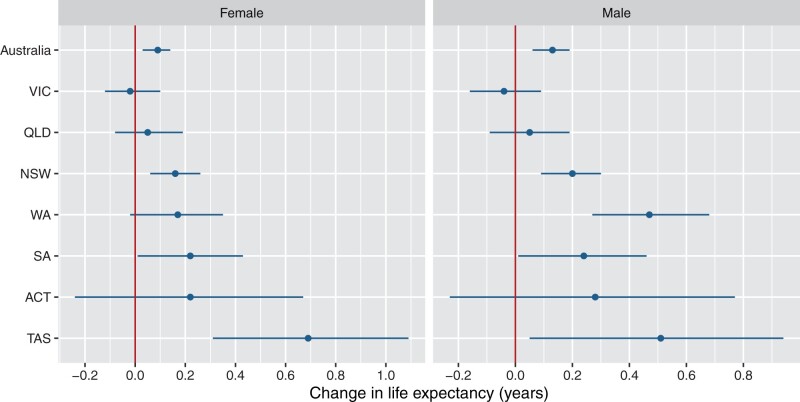
Change in life expectancy at birth by state/territory and sex, Australia, 2017–19 to 2020–22. Bars show 95% confidence intervals and the vertical line (red) corresponds to no change in life expectancy compared with 2017–19. Northern Territory is not presented in the figure because it had a very high level of late registration in 2022. VIC, Victoria; QLD, Queensland; NSW, New South Wales; WA, Western Australia; SA, South Australia; ACT, Australian Capital Territory; TAS, Tasmania. Source: author calculations based on Australian Bureau of Statistics data^14–16^^,31^

**Figure 6.**
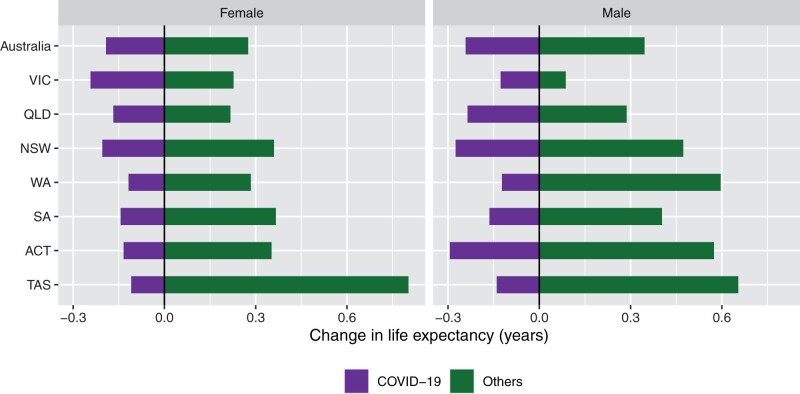
Cause decomposition of the change in life expectancy at birth by state/territory and sex, Australia, 2017–19 to 2020–22. Northern Territory is not presented in the figure because it had a very high level of late registration in 2022. VIC, Victoria; QLD, Queensland; NSW, New South Wales; WA, Western Australia; SA, South Australia; ACT, Australian Capital Territory; TAS, Tasmania. Source: author calculations based on Australian Bureau of Statistics data^9^^,^^14–16^^,31^

There was a reasonable degree of uniformity in annual life expectancy change in the states and territories during 2020–22 (Figures 5–6; Supplementary Figures S4 and S5, available as Supplementary data at *IJE* online for annual change). In Victoria, which had the most COVID-19 deaths and the most stringent restrictions, life expectancy fell very slightly from 2017–19 to 2020–22 but with the upper 95% confidence interval overlapping with zero (females: −0.02 years, −0.12 to 0.10; males: −0.04 years, −0.15 to 0.08). The contribution of COVID-19 to life expectancy change in Victoria was −0.24 years for females and −0.13 years for males. NSW had a similar change in life expectancy during 2020–22 as for all of Australia, with the contribution of COVID-19 being only slightly greater than in all of Australia. In other states, where restrictions were not as strict as in Victoria and NSW, the change in life expectancy over the pandemic ranged from slightly lower than Australia in Queensland, although with overlapping confidence intervals, to higher in South Australia, WA (especially for males) and Tasmania, with often wide confidence intervals. In these states, the contribution of COVID-19 was lower than in Australia as a whole. In 2022 specifically, declines in life expectancy were relatively large in males in the ACT (with wide confidence intervals).

Actual life expectancy in Australia in 2020 was higher than forecast (0.31 years females, 0.20 males) but the gap was higher when compared with actual life expectancy in 2017–19 (Table 1). In 2021, the deficit was lower than the surplus in 2020. In 2022, actual life expectancy was approximately 1 year below that forecast, of which 0.36 years for females and 0.42 years for males were due to COVID-19. Over 2020 to 2022, life expectancy was 0.36 years lower than forecast for males and 0.25 years lower for females, of which the majority was due to COVID-19.

**Table 1.** Comparison of forecast (Lee–Carter using 2010–19 data) and actual life expectancy by sex and year(s), Australia

Sex and year(s)	**Lee**–**Carter forecast (80% CI)**	Actual (80% CI)	Difference and cause contribution		
			Difference	COVID-19	Other causes
Female					
2020	85.35 (85.01-85.63)	85.66 (85.59-85.74)	+0.31	–0.05	+0.36
2021	85.45 (85.00-85.85)	85.34 (85.27-85.42)	–0.11	–0.01	–0.10
2022	85.56 (85.01-86.09)	84.66 (84.59-84.73)	–0.90	–0.36	–0.54
2020–22	85.45 (85.01-85.86)	85.20 (85.16-85.24)	–0.25	–0.19	–0.06
Male					
2020	81.43 (81.18-81.64)	81.63 (81.56-81.71)	+0.20	–0.05	+0.25
2021	81.58 (81.22-81.87)	81.43 (81.35-81.50)	–0.14	–0.04	–0.11
2022	81.73 (81.29-82.13)	80.67 (80.59-80.74)	–1.06	–0.42	–0.64
2020–22	81.58 (81.23-81.88)	81.22 (81.18-81.26)	–0.36	–0.24	–0.12

Source: author’s calculations based on Human Mortality Database and Australian Bureau of Statistics data.^14–16^^,31^ CI, confidence interval.

## Discussion

Australia’s life expectancy has fared well relative to that in other high-income and upper middle-income countries during the COVID-19 pandemic. Following a large increase in 2020, it returned to a more normal trajectory in 2021. Although life expectancy declined by more than half a year in 2022, the largest annual decline in several decades, it was marginally higher over 2020–22 than over 2017–19 because of the gains made in 2020 and 2021.^35^ When compared with forecast life expectancy, it was almost 1 year lower in 2022 but only slightly lower over the period 2020–22. Although this decline in 2022 may appear small given that excess mortality for that year was measured as 12%, this reflects the relatively old ages at which excess deaths occurred.^13^ During 2020, Australia had more favourable changes in life expectancy than comparator countries except New Zealand, and in that year it would have had more than double the deaths if it had Bulgaria’s or Hungary’s death rates and 65% more deaths if it had the USA’s death rates. However, its decline in life expectancy in 2022 was greater than for other countries whose life expectancy had increased in the first year of the pandemic.

COVID-19 deaths in Australia were minimal in 2020 and 2021, contributing less than 0.10 years to life expectancy decline during a period when international borders were closed and governments introduced relatively strict lockdowns to control the virus. In 2022, when restrictions were significantly relaxed, COVID-19 mortality increased substantially and contributed to most of the declines in life expectancy that year. Over the period 2020–22 however, COVID-19 only contributed to a decline in life expectancy of about 0.2 years. The reversal of its contribution to the increase in life expectancy in 2020 was most pronounced among the 80 years and above age group, including in 2021 and especially 2022 when COVID-19 had the greatest impact on mortality. In addition to COVID-19, dementia, diabetes and external and other causes (combined) also contributed to declines in life expectancy over 2020–22, but to a lesser extent. For dementia and diabetes, this may simply be a continuation of longer-term increases in mortality from these causes relative to other causes.^9^ ‘Other’ causes made a particularly significant negative contribution to life expectancy in 2021. Published ABS total deaths from each cause show that several causes likely to disproportionately affect older people—such as Parkinson’s disease, disorders of gallbladder, biliary tract and pancreas, diseases of the musculoskeletal system and connective tissue and other disorders of urinary system—had annual increases in deaths in 2021 of at least 10%; this is consistent with the majority of decline in life expectancy in 2021 due to ‘other’ causes being due to deaths in ages 80+ years (Supplementary Figure S3, available as Supplementary data at *IJE* online).^9^ Cancers, ischaemic heart disease and cerebrovascular diseases made a positive contribution to life expectancy over the 3 years to the end of 2022, especially in 2020.

Despite Victoria having a higher COVID-19 death rate than other states and territories in 2020–21, both for these years and over 2020–22, it experienced only a slight decrease in life expectancy, which had an upper 95% confidence interval overlapping with zero (i.e. no change). In NSW, with the next most COVID-19 deaths, life expectancy trends were almost identical to Australia. In other states and territories, there was some variation in life expectancy over the pandemic, although not substantial.

Australia’s slightly higher life expectancy for the period 2020–2022 compared with 2017–19 demonstrates its relatively favourable mortality outcome during the pandemic. This can be attributed to several factors which have implications for policy makers in other countries, including the prompt closure of international borders in March 2020, relatively stringent lockdowns and opening up of most restrictions only once vaccination rates had reached a high level. The relatively similar life expectancy trends of Victoria and NSW with the rest of Australia is testament to the effectiveness of lockdowns in these locations where outbreaks of the virus were worse. Compared with countries with much worse life expectancy outcomes during the pandemic, such as Bulgaria and the USA, interventions such as lockdowns and vaccine roll-outs were clearly more effective in Australia.^38^^,^^39^ Among countries that experienced increases in life expectancy in 2020—New Zealand, Norway, Finland and Denmark—relatively stringent restrictions were also found, as in Australia.^40^^,^^41^ These countries, also like Australia, thereafter mostly experienced declines in life expectancy in 2021 and 2022 (where data were available). In Sweden, which had less stringent restrictions than its Nordic neighbours, life expectancy fell in 2020 but then rose in the next 2 years.^42^ The findings suggest that over the first 3 years of the pandemic, initial trends in mortality in these countries were to some extent offset by displacement of mortality that is commonly identified during pandemics and other mortality shocks.^43^^,^^44^

For Australia, there had been a concern that, following reduced circulation of respiratory infections in 2020–21, the loosening of restrictions would lead to influenza and pneumonia deaths increasing which would adversely affect older and more frail populations because of mortality displacement. However, deaths from respiratory causes in 2022 only contributed to a slight decline in life expectancy, and influenza deaths were much lower than in some previous years (e.g. only 287 in 2022 compared with over 1250 in 2017).^9^^,^^15^ From 2020 to 2022, respiratory diseases contributed to an increase in life expectancy almost equal to the decline due to COVID-19, also when disaggregated by age and sex, suggesting it was somewhat due to people dying from COVID-19 who possibly had pneumonia as a more proximate cause or had a pre-existing chronic respiratory disease. Given that the majority of life expectancy decline in 2021 was due to increased mortality of those at 80+ years, primarily due to ‘other’ causes, it is likely that some of this mortality displacement occurred in that year. The positive contribution of cancers, ischaemic heart disease and cerebrovascular diseases to life expectancy over the pandemic to 2022, especially in 2020, allays some fears that reduced access to health services due to restrictions may have adversely affected mortality from these causes.

A limitation of the study was that only a set of 11 causes of death were obtained from the ABS. This meant that a residual category of ‘other’ causes was included in the analysis and for which we can only estimate the major contributory causes although, as described above for 2021 data, available ABS published cause totals can be used to understand the likely contributory specific causes to this cause category. Furthermore, as mentioned in the Methods section and the Supplementary Material (available as Supplementary data at *IJE* online), we had to adjust data for late registration in 2022, although this only increased total deaths by 0.6% nationally and we did not include adjusted estimates for the Northern Territory which had a high level of late registration. There were also only doctor-certified death data available for 2022; however, coroner-certified deaths were only 13% of all deaths, and external causes—the cause group with the highest proportion of coroner-certified deaths—we combined with ‘other’ causes to reduce the risk of inaccurate estimates. Another limitation is that the metric of life expectancy can underestimate the impact of changes in mortality at the oldest ages, which is relevant in 2022 when the decline in life expectancy appears small compared with excess mortality for that year. However, the trends in life expectancy at 60 years were very similar to life expectancy at birth. Finally, the study was reliant upon data of the underlying cause of death; analysis of multiple cause of death data would reveal whether more proximate causes may have affected these trends, such as pneumonia as a proximate cause of dementia given the susceptibility of older populations to respiratory infections. Multiple cause data would also show comorbidities of COVID-19, such as diabetes and cardiovascular diseases.

## Conclusion

The findings from this study provide a valuable international case study into life expectancy trends in a country during a period with stringent restrictions and low COVID-19 mortality and then following loosening of these restrictions. The study has shown how an analysis of the whole pandemic to date is worthwhile because annual trends can tend to offset previous changes due to mortality displacement. Longer term, there may be impacts on mortality of new variants of the virus, the level of ongoing vaccination coverage, long COVID, and reductions in screening of various medical conditions during the pandemic.

## Ethics approval

Ethics approval was not required because the study is an analysis of aggregate data compiled by the Australian Bureau of Statistics.

## Supplementary Material

dyad121_Supplementary_Data

## Data Availability

R code and data are available at [https://github.com/bhoule13/au-ex2022].
