# Supplementary material for: Effect of the COVID-19 pandemic on life expectancy in Australia, 2020-22
Source: Int J Epidemiol. 2023 Sep 25;52(6):1735–44. doi: 10.1093/ije/dyad121 (PMC10749770; doi:10.1093/ije/dyad121)
Supplement: dyad121_Supplementary_Data [file dyad121_supplementary_data.docx]

**Supplementary Material**

**Additional information about data sources and methods**

For 2017-20, we used all-cause mortality data according to year of occurrence published by the Australian Bureau of Statistics (ABS).^1^ For these same years we used published cause of death data from the ABS by year of occurrence and sex; these data were not published by detailed age at death, so we applied the proportion of deaths for each year, sex and cause that occurred in each age in Australia from the World Health Organization (WHO) Mortality Database (by year of registration) to the ABS’s total deaths for each year, sex and cause.^2, 3^ All-cause mortality data for deaths that occurred in 2021 and 2022 were purchased by the authors from the ABS.^4, 5^ We assumed no late registration of 2021 deaths, but adjusted 2022 deaths for late registration using monthly reports of deaths by the ABS in 2022; we calculated the average proportional increase in reported deaths according to the number of months since occurrence, by sex and broad age group, which inflated total 2022 deaths by 0.6%.^6-17^

Due to 2022 cause of death data only being available for the 87% of deaths that were doctor-certified, we estimated cause-specific coroner- certified deaths in 2022 by applying the cause distribution of age-sex-specific coroner-certified deaths in 2021 to the age-sex-specific coroner-certified deaths in 2022: ^5^

$\tilde{D}_{sxjc2}=D_{sxc2}*\frac{D_{sxjc1}}{D_{sxc1}}$, (1)

where $\tilde{D}$ and *D* are estimated and reported deaths respectively, *s* is sex, *x* is age, *j* is cause, *c* is coroner-certified, d is doctor-certified, time *1* is 2021 and time *2* is 2022. Further, the total estimated cause-specific deaths (doctor and coroner-certified) are simply:

${\tilde{D}_{sxj2}=D_{sxjd2}+\tilde{D}}_{sxjc2}$. (2)

After summing the reported doctor-certified and estimated coroner-certified cause-specific deaths for 2022 to calculate the estimate of the total cause-specific deaths for that year, we then separately adjusted age-specific COVID-19 deaths (doctor- plus coroner-certified, as estimated in equation 2) using a sex-specific adjustment factor to ensure they equalled the total sex-specific COVID-19 deaths (doctor- plus coroner-certified) reported by the ABS for 2022: ^18^

$\hat{D}_{sxv2}=\tilde{D}_{sxv2}N_{s}$ , (3)

where $\hat{D}_{sxv2}$ and $\tilde{D}_{sxv2}$ are the final and eq(2) estimated deaths for sex *s*, at age *x*, and cause of death *v* = COVID-19 at time 2, respectively, and $N_{s}= \frac{D_{sv2}}{\sum_{x} \tilde{D}_{sxv2}}$ is the sex-specific adjustment factor that is constant across age, with $D_{sv2}$ the ABS COVID-19 published total deaths for sex *s* at time 2.

Sex-specific deaths from each other cause were reduced proportionally (with a constant percentage reduction for each age) so that the sum of cause-specific deaths equalled total all-cause deaths.

State and territory all-cause mortality data were obtained from the same sources as for Australia.^1, 2, 4, 5^ State and territory death data for 2022 were adjusted for late registration again using ABS reports of monthly deaths in 2022, but they were only available for all ages and both sexes; these were hence calculated using state/territory data for all ages and both sexes and national data by age and sex, and then further adjusted to ensure state/territory total deaths added to national total deaths. Analyses including 2022 data in Northern Territory were not presented in this study because it had a very high level of late registration in that year.

We adjusted doctor-certified COVID-19 deaths by age and sex so that they equalled the ABS-published total COVID deaths by state/territory and sex: ^18, 19^

$\hat{D}_{sxiv2}=D_{sxivd2} N_{si}$, (4)

where *i* is state/territory and *d* is doctor-certified, and $N_{si}=\frac{D_{siv2}}{\sum_{x} D_{sxivd2}}$. Sex-specific ‘other’ causes of death were reduced proportionally (with a constant percentage reduction for each age) so that the sum of cause-specific deaths equalled total all-cause deaths.

**Additional figures and tables**

**Figure S1: Change in life expectancy by sex and year(s), Australia**

Note: Bars show 95% confidence intervals and the red line corresponds to no change in life expectancy compared with 2017-19.

Source: Author’s calculations based on ABS data. ^1, 4, 5, 20^

**Table S1: Life expectancy at 60 years and change by sex and year(s), Australia**

|  | **Female**  **(lower to upper 95% CI)** | **Male**  **(lower to upper 95% CI)** |
| --- | --- | --- |
| **Life expectancy at 60 years** |  |  |
| 2017-19 | 27.14 (27.11 to 27.17) | 24.31 (24.27 to 24.34) |
| 2020 | 27.63 (27.57 to 27.69) | 24.76 (24.70 to 24.82) |
| 2021 | 27.32 (27.27 to 27.37) | 24.54 (24.48 to 24.59) |
| 2022 | 26.73 (26.67 to 27.73) | 23.85 (23.80 to 23.90) |
| 2020-22 | 27.20 (27.17 to 27.23) | 24.36 (24.33 to 24.39) |
| **Change in life expectancy at 60 years** |  |  |
| 2017-19 to 2020 | +0.49 (+0.43 to +0.55) | +0.45 (+0.39 to +0.52) |
| 2020 to 2021 | -0.31 (-0.39 to -0.23) | -0.22 (-0.30 to -0.14) |
| 2021 to 2022 | -0.59 (-0.67 to -0.52) | -0.69 (-0.77 to -0.62) |
| 2017-19 to 2020-22 | +0.06 (+0.02 to +0.11) | +0.05 (+0.01 to +0.09) |

Source: Author’s calculations based on HMD and ABS data. ^1, 4, 5, 21^ CI: confidence interval.

**Table S2: Number of deaths and difference (number and %) estimated to have occurred in Australia in 2020 assuming the same death rates as other countries**

| **Country** | **Female** | | | **Male** | | | | **Both sexes** | | | |
| --- | --- | --- | --- | --- | --- | --- | --- | --- | --- | --- | --- |
|  | **Deaths** | **Diff. (n)** | **Diff. (%)** | **Deaths** | **Diff. (n)** | | **Diff. (%)** | **Deaths** | | **Diff. (n)** | **Diff. (%)** |
| Australia | 77,372 | - | - | 85,194 | - | | - | 162,566 | | - | - |
| Hong Kong | 59,492 | -17,880 | -23.1 | 81,365 | -3,829 | | -4.5 | 140,346 | | -22,220 | -13.7 |
| Japan | 61,417 | -15,955 | -20.6 | 88,447 | +3,253 | | +3.8 | 146,643 | | -15,923 | -9.8 |
| Republic of Korea | 72,478 | -4,894 | -6.3 | 97,726 | +12,532 | | +14.7 | 165,033 | | +2,467 | +1.5 |
| New Zealand | 87,073 | +9,701 | +12.5 | 91,852 | | +6,658 | +7.8 | | 178,765 | +16,198 | +10.0 |
| Norway | 88,082 | +10,710 | +13.8 | 91,881 | | +6,686 | +7.8 | | 179,281 | +16,714 | +10.3 |
| Iceland | 92,499 | +15,127 | +19.6 | 89,895 | | +4,701 | +5.5 | | 183,045 | +20,479 | +12.6 |
| France | 81,292 | +3,920 | +5.1 | 106,997 | | +21,802 | +25.6 | | 184,846 | +22,279 | +13.7 |
| Switzerland | 88,492 | +11,120 | +14.4 | 98,183 | | +12,988 | +15.2 | | 184,993 | +22,427 | +13.8 |
| Canada | 88,415 | +11,043 | +14.3 | 100,461 | | +15,266 | +17.9 | | 188,338 | +25,771 | +15.9 |
| Ireland | 92,100 | +14,728 | +19.0 | 98,669 | | +13,475 | +15.8 | | 190,498 | +27,932 | +17.2 |
| Spain | 87,832 | +10,460 | +13.5 | 110,267 | | +25,073 | +29.4 | | 195,382 | +32,816 | +20.2 |
| Finland | 90,085 | +12,713 | +16.4 | 109,823 | | +24,629 | +28.9 | | 197,189 | +34,622 | +21.3 |
| Sweden | 95,478 | +18,106 | +23.4 | 102,718 | | +17,524 | +20.6 | | 197,545 | +34,979 | +21.5 |
| Luxembourg | 92,761 | +15,389 | +19.9 | 109,192 | | +23,998 | +28.2 | | 200,081 | +37,514 | +23.1 |
| Denmark | 99,174 | +21,802 | +28.2 | 110,183 | | +24,989 | +29.3 | | 208,024 | +45,458 | +28.0 |
| Portugal | 94,777 | +17,405 | +22.5 | 121,560 | | +36,366 | +42.7 | | 211,530 | +48,963 | +30.1 |
| Germany | 102,218 | +24,846 | +32.1 | 118,753 | | +33,559 | +39.4 | | 218,604 | +56,038 | +34.5 |
| England & Wales | 107,451 | +30,079 | +38.9 | 116,760 | | +31,565 | +37.1 | | 223,540 | +60,974 | +37.5 |
| Northern Ireland | 112,426 | +35,054 | +45.3 | 119,166 | | +33,971 | +39.9 | | 230,264 | +67,698 | +41.6 |
| Chile | 116,177 | +38,805 | +50.2 | 138,766 | | +53,572 | +62.9 | | 251,114 | +88,547 | +54.5 |
| Scotland | 125,176 | +47,804 | +61.8 | 136,747 | | +51,552 | +60.5 | | 260,251 | +97,684 | +60.1 |
| U.S.A. | 124,731 | +47,359 | +61.2 | 144,006 | | +58,812 | +69.0 | | 267,404 | +104,837 | +64.5 |
| Czechia | 132,348 | +54,976 | +71.1 | 165,257 | | +80,063 | +94.0 | | 290,612 | +128,046 | +78.8 |
| Croatia | 137,950 | +60,578 | +78.3 | 168,154 | | +82,959 | +97.4 | | 299,217 | +136,651 | +84.1 |
| Lithuania | 135,351 | +57,978 | +74.9 | 209,452 | | +124,258 | +145.9 | | 324,397 | +161,830 | +99.5 |
| Hungary | 148,305 | +70,933 | +91.7 | 191,007 | | +105,812 | +124.2 | | 328,438 | +165,872 | +102.0 |
| Bulgaria | 172,264 | +94,891 | +122.6 | 228,506 | | +143,312 | +168.2 | | 390,739 | +228,173 | +140.4 |

Countries sorted by difference for both sexes. Diff.: Difference. n: Number.

Source: Author’s calculations based on ABS and HMD data. ^1, 4, 5, 20, 21^

**Figure S2: Contribution of detailed causes of death to change in life expectancy by sex and year(s), Australia**


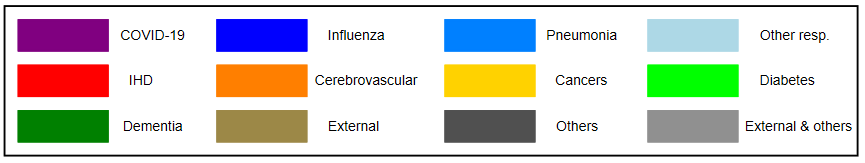


Note: Bars to the left (negative) corresponds to contributions to decline in life expectancy, while to the right (positive) to increase. Only a relatively small proportion of external cause deaths are certified by doctors. The method to estimate cause specific deaths by adjusting doctor-certified deaths to all deaths (doctor-certified plus coroner-certified) in 2022 would hence result in large uncertainty in estimated external cause deaths. To reduce this uncertainty, external causes and other causes were combined in 2022. ICD codes for dementia were F01, F03 and G30, diabetes E10-E14 and external causes V01-Y98.

Source: Author’s calculations based on ABS and WHO data. ^1-5, 20^

**Figure S3: Contribution of age group and causes of death to change in life expectancy by sex and year(s), Australia**

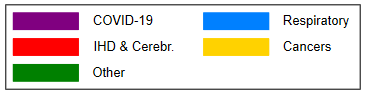


Bars to the left (negative) corresponds to contributions to decline in life expectancy, while to the right (positive) to increase.

Source: Author’s calculations based on ABS and WHO data. ^1-5, 20^

**Figure S4: Change in life expectancy by sex, year(s) and state/territory, Australia, 2017-19 to 2022**

Note: Bars show 95% confidence intervals and the red line corresponds to no change in life expectancy compared with 2017-19. Northern Territory is not presented in the Figure because it had a very high level of late registration in 2022.

Source: Author’s calculations based on ABS data. ^1, 4, 5, 20^

**Figure S4 (contd.): Change in life expectancy by sex, year(s) and state/territory, Australia, 2017-19 to 2022**

Note: Bars show 95% confidence intervals and the red line corresponds to no change in life expectancy compared with 2017-19. Northern Territory is not presented in the Figure because it had a very high level of late registration in 2022.

Source: Author’s calculations based on ABS data. ^1, 4, 5, 20^

**Figure S5: Cause decomposition of the change in life expectancy change by sex, year(s) and state/territory, Australia, 2017-19 to 2022**

**Figure S5 (contd.): Cause decomposition of the change in life expectancy change by sex, year(s) and state/territory, Australia, 2017-19 to 2022**

Note: Northern Territory is not presented in the Figure because it had a very high level of late registration in 2022.

Source: Author’s calculations based on ABS data. ^1, 2, 4, 5, 20^

**References**

1. Australian Bureau of Statistics. *Deaths, Australia, 2021*. Canberra: Australian Bureau of Statistics; 2022.

2. Australian Bureau of Statistics. *Causes of Death, Australia, 2021*. Canberra: Australian Bureau of Statistics; 2022.

3. World Health Organization. WHO Mortality Database. Geneva; 2022.

4. Australian Bureau of Statistics. *Provisional Mortality Statistics: Customised Report*. Canberra: Australian Bureau of Statistics; 2022.

5. Australian Bureau of Statistics. *Provisional Mortality Statistics: Customised Report*. Canberra: Australian Bureau of Statistics; 2023.

6. Australian Bureau of Statistics. *3303.0.55.004 Provisional Mortality Statistics, Australia, Jan 2022*. Canberra: Australian Bureau of Statistics; 2022.

7. Australian Bureau of Statistics. *3303.0.55.004 Provisional Mortality Statistics, Australia, Jan - Feb 2022*. Canberra: Australian Bureau of Statistics; 2022.

8. Australian Bureau of Statistics. *3303.0.55.004 Provisional Mortality Statistics, Australia, Jan - Mar 2022*. Canberra: Australian Bureau of Statistics; 2022.

9. Australian Bureau of Statistics. *3303.0.55.004 Provisional Mortality Statistics, Australia, Jan - Apr 2022*. Canberra: Australian Bureau of Statistics; 2022.

10. Australian Bureau of Statistics. *3303.0.55.004 Provisional Mortality Statistics, Australia, Jan - May 2022*. Canberra: Australian Bureau of Statistics; 2022.

11. Australian Bureau of Statistics. *3303.0.55.004 Provisional Mortality Statistics, Australia, Jan - Jun 2022*. Canberra: Australian Bureau of Statistics; 2022.

12. Australian Bureau of Statistics. *3303.0.55.004 Provisional Mortality Statistics, Australia, Jan - Jul 2022*. Canberra: Australian Bureau of Statistics; 2022.

13. Australian Bureau of Statistics. *3303.0.55.004 Provisional Mortality Statistics, Australia, Jan - Aug 2022*. Canberra: Australian Bureau of Statistics; 2022.

14. Australian Bureau of Statistics. *3303.0.55.004 Provisional Mortality Statistics, Australia, Jan - Sep 2022*. Canberra: Australian Bureau of Statistics; 2022.

15. Australian Bureau of Statistics. *3303.0.55.004 Provisional Mortality Statistics, Australia, Jan - Nov 2022*. Canberra: Australian Bureau of Statistics; 2023.

16. Australian Bureau of Statistics. *3303.0.55.004 Provisional Mortality Statistics, Australia, Jan - Dec 2022*. Canberra: Australian Bureau of Statistics; 2023.

17. Australian Bureau of Statistics. *3303.0.55.004 Provisional Mortality Statistics, Australia, Jan 2023*. Canberra: Australian Bureau of Statistics; 2023.

18. Australian Bureau of Statistics. *COVID-19 Mortality in Australia: Deaths registered until 31 March 2023*. Canberra: Australian Bureau of Statistics; 2023.

19. Australian Bureau of Statistics. *COVID-19 Mortality by wave*. Canberra: Australian Bureau of Statistics; 2022.

20. Australian Bureau of Statistics. *National, state and territory population, June 2022*. Canberra: Australian Bureau of Statistics; 2022.

21. Human Mortality Database. Max Planck Institute for Demographic Research (Germany), University of California, Berkeley (USA), and French Institute for Demographic Studies (France). https://www.mortality.org/ (7 March 2023).
